# The effect of different adhesive strategies and diamond burs on dentin bond strength of universal resin cements

**DOI:** 10.1007/s00784-024-06112-4

**Published:** 2025-01-03

**Authors:** Chavakorn Atsavathavornset, Pipop Saikaew, Choltacha Harnirattisai, Hidehiko Sano

**Affiliations:** 1 Department of Operative Dentistry and Endodontics, Faculty of Dentistry, Mahidol University, No. 6, Yothi Road, Ratchathewi District, Bangkok, 10400 Thailand; 2 Department of Restorative Dentistry, Graduate School of Dental Medicine, Hokkaido University, Sapporo, Japan

**Keywords:** Adhesive strategies, Self-adhesive resin cement, Smear layer, Universal adhesive, Universal resin cement

## Abstract

**Objectives:**

To evaluate the shear bond strength (SBS) of universal cements (UCs) to dentin prepared with different diamond burs using various adhesive strategies.

**Materials and methods:**

One-hundred-twenty molars were prepared to expose the mid-coronal dentin. The teeth were divided into two groups according to diamond bur preparations: coarse and super-fine grit burs. The specimens were bonded to lithium disilicate discs using two UCs (RelyX Universal, RXU and Panavia SA Luting Multi, PSA) with different adhesive strategies (self-adhesive, SA; self-etch, SE and etch-and-rinse, ER). SBS was measured using a universal testing machine. The cement-dentin interfaces were observed using scanning electron microscopy.

**Results:**

Dentin SBS was significantly influenced by the adhesive strategies and the type of UCs (*p* < 0.05) but not for the different diamond bur preparations (*p* > 0.05). UCs used with ER had significantly higher SBS values than UCs used in SA mode (*P* < 0.05), except for PSA with super-fine diamond bur. RXU in SE mode exhibited significantly higher SBS than SA mode (*P* < 0.05). Regarding UCs, RXU showed a higher SBS than PSA, particularly in the SE modes when the dentin was prepared with a coarse diamond bur (*P* < 0.05).

**Conclusion:**

The use of UCs combined with universal adhesives exhibited higher dentin bonding performance compared with the use of UCs alone.

**Clinical significance:**

The etch-and-rinse mode combined with a universal adhesive is recommended to achieve the highest dentin bond strength of universal cements.

## Introduction

Resin cement has become increasingly popular for cementing indirect restorations due to its lower solubility and greater esthetics than conventional resin cement [1–3] that provide strong and predictable long-term bonding to tooth tissues. Resin cement can be classified into three groups: etch-and-rinse, self-etch resin cement and self-adhesive resin cement [4].

The conventional resin cements, including the etch-and-rinse and self-etch groups, have demonstrated high clinical performance. However, the adhesive luting procedure is complex and technique sensitive [5]. Therefore, self-adhesive resin cements (SARCs) were developed to simplify the luting procedure. Unlike conventional resin cements that require multiple steps, SARCs can be bonded to dentin or enamel without adhesive application. Their application can be completed in one step, making them popular in clinical use. Furthermore, it was reported that SARCs have shown good clinical performance when restored with partial indirect restorations in both the short term [6] (at least 1 year) and long term [7] (15 years), comparable to conventional resin cements. However, laboratory studies have reported compromised bond strength of SARCs compared with conventional resin cements that involve separate steps of adhesive application [8].

Previous studies demonstrated that the quality of adhesion of SARCs to dentin and enamel can be improved with the use of adhesive systems [9–12]. Moreover, a study demonstrated that applying a bonding agent enhanced the immediate dentin bond strength of SARCs and long-term survival rates [9]. According to a recent classification [13], self-adhesive cements that can be used optionally with universal adhesives are classified as universal cements. Therefore, this cement is as simple as SARC and versatile in different clinical situations.

Tooth preparation is regularly performed with diamond burs, which usually creates a smear layer on the dentin surface. It was reported that the preparation methods and diamond bur grain size can affect the characteristics of the smear layer [14]. The use of diamond burs creates a dense smear layer, while SiC paper preparation results in a non-homogeneous smear layer. Moreover, it has been demonstrated that the smear layer thickness is dependent on the size of the abrasive particles used. Greater abrasive particle size results in a thicker smear layer [14–16]. Several studies have investigated the impact of various dentin preparation methods on the bond strength of resin cements [17–18]. One study reported the higher immediate bond strength of etch-and-rinse resin cement compared with SARCs, regardless of the dentin preparation method [17]. Another study showed a significantly higher bond strength of dentin prepared with superfine-grit polishing discs compared with those prepared with a fine-grit diamond bur when self-etch resin cement and SARC were used [18]. Similarly, the use of a superfine-grit diamond bur significantly enhanced the bond strength of self-etch resin cement to dentin, compared with that of regular-grit diamond burs [19–20].

Currently, there is no report on the effect of adhesive strategies of universal resin cements (UCs) on bur-cut smear layers. Therefore, the objective of this study was to evaluate the bond strength between different adhesive strategies on UCs to dentin prepared with different diamond burs. The null hypothesis was (1) There was no significant difference in the dentin bond strength of UCs with different adhesive strategies. (2) There was no significant difference in the bond strength of UCs bonded to dentin prepared using diamond burs with different particle sizes. (3) There was no significant difference in the dentin bond strength of different type of UCs.

## Materials and methods

### Tooth collection and tooth preparation

In this study, 132 extracted human third molars without carious lesions, cracks, and restorations on the enamel and dentin surface were used and collected under a protocol that was approved by the University Ethics Committee. The sample size was calculated from the estimated effect size between adhesive strategies and dental burs in a pilot study (95% power and 5% of error) *N* = 10 (G*Power 3.1) [21]. The collected teeth were stored in a 0.1% thymol solution and used within 6 months after extraction. One-hundred and twenty teeth were cut perpendicular to their long axis using a low-speed diamond saw (Diamond blade 4-inch series HC, PACE, USA) until the mid-coronal dentin was exposed. The teeth were then embedded in cylindrical polyvinyl chloride rings (2 cm diameter) using chemically cured acrylic resin. The specimens were randomly divided into two groups based on the abrasive particle size of the diamond burs [19] (coarse, 852G.016, 107–181 μm particle size, ISO534; superfine, 852EF.016, 10–36 μm particle size ISO504, diamond point FG; Jota, Rüthi, Switzerland). A single operator was trained and calibrated how to apply 100 g of pressure prior to performing the surface preparation [22]. The dentin surfaces were ground using the burs mounted in a high-speed handpiece with copious water spray for 5 light-pressure strokes. The bur was replaced after 5 samples were prepared [22].

### Adhesives and cementation procedure

The dentin was dried with gauze and then blown with triple syringe air for 5 s before the cementation step. The specimens were further divided into six subgroups (*n* = 10) according to UCs that describe in Table 1 (Rely X Universal, RXU; Panavia SA Luting Multi, PSA) and adhesive strategies (no treatment; SA, self-etch: SE, etch-and-rinse; ER).

**Table 1:** List of materials and their composition

Materials	Type of material	Manufacturer and Lot number	Composition
Rely X Universal (RXU)	Resin cement	3 M ESPE, USA 10049940	paste A: DUDMA, TEGDMA, 2-propenoic acid 2 methyl- 3-(trimethoxysilyl)propyl ester reaction products with various silica, mixture of mono-di- and tri-glycerol dimethacrylate ester of phosphoric acid, silane trimethoxyoctyl hydrolysis products with silica, t-amyl hydroperoxide, 2,6-di-tert-butyl-p-cresol, HEMA, methyl methacrylate, acetic acid copper(2+) salt monohydrate Paste B: DUDMA, TEGDMA, ytterbium (III) fluoride, glass powder surface modified with 2-propenonic acid, 2-methyl-3-(trimethoxysilyl)propyl ester and phenyltrimethoxy silane bulk material, L-ascorbic acid 6-hexadecanoate hydrate, silane trimethoxyoctyl- hydrolysis products with silica, titanium dioxide, triphenyl phosphite
Single Bond Universal Plus (SBUP)	Universal adhesive	3 M ESPE, USA 10042324	Primer: MDP, HEMA, Vitrebond copolymer, brominated dimethacrylate resins (BPA derivative-free), ethanol, water, initiators, dual-cure accelerator, optimized mixture of silane, filler
Panavia SA Luting Multi (PSA)	Resin cement	Kuraray Noritake, Japan 140220	MDP, Bis-GMA, TEGDMA, HEMA, methacrylate monomer, other methacrylate monomers, silanated barium glass filler, silanated colloidal silica, initiator, aluminum oxide, silanated sodium fluoride, accelerator, pigment, silane coupling agent, others
Clearfil Tri-s Bond Universal Quick (CBQ)	Universal adhesive	Kuraray Noritake, Japan 8G0370	Primer: Bis-GMA, MDP, HEMA, hydrophilic amide monomer, filler, ethanol, water, NaF, photo initiators, chemical polymerization accelerator, silane coupling agent, others
3M^TM^ Scotchbond^TM^ Universal Etchant	37% Phosphoric acid etchant gel	3 M ESPE, USA 9984134	Water, Phosphoric Acid, Synthetic Amorphous Silica, Fumed, Crystalline Free Polyethylene Glycol, Aluminium Oxide
Porcelain etchant gel	Porcelain etchant gel	Pulpdent	9.6% Hydrofluoric Acid in a proprietary gel base
Monobond N	Universal Primer	Ivoclar-Vivadent, Schann, Liechtenstein Z05HH3	Ethanol, water, 3-MPS, 10-MDP and 10-MDDT

Abbreviation: DUDMA, Diurethane dimethacrylate; TEGDMA, Triethylene glycol dimethacrylate; HEMA, hydroxy ethyl methacrylate; MDP, methacryloyloxydecyl dihydrogen phosphate; Bis-GMA, bisphenol A-glycidyl methacrylate; 3-MPS, γ-Methacryloyloxypropyl trimethoxysilane; 10-MDDT, 10-methacryloyloxydecyl 6,8-dithiooctanoate

The cementation technique was applied according to the manufacturer’s instructions as described in Table 2 without curing the adhesive layer. Lithium di-silicate cylinder discs (IPS.eMax CAD, HT Shade A2, Ivoclar-Vivadent, Schann, Liechtenstein), were fabricated by milling lithium disilicate blocks into cylinders. The cylinders were then crystallized in a furnace (VITA V60 i-Line PLUS) and cut into 3.6 mm diameter and 2 mm thick discs [23]. The ceramic discs were treated with 9.6% hydrofluoric acid gel (Porcelain etchant gel, Pulpdent, USA) for 20 s. The specimens were rinsed and cleaned in an ultrasonic machine for 5 min, followed by applying Universal primer (monobond n, Ivoclar-Vivadent, Schann, Liechtenstein), and air-dried after being left on the ceramic surface for 60 s.

**Table 2:** Adhesive strategies and application of materials

System	Adhesive strategies	Materials	Application instructions
RXU	SA	Rely X Universal	1. Apply with an auto-mix tip (provided by manufacturer), light cure for 10 s
	SE	Single Bond Universal Plus, Rely X Universal	1. Apply Single bond universal plus to the dentin surface and rub it for 20 s: gentle air dry for 5 s without light curing 2. Apply cement with an auto-mix tip, light cure for 10 s
	ER	37% Phosphoric acid etchant gel, Single Bond Universal Plus, Rely X Universal	1. Apply etchant gel to dentin for 15 s; rinsed with distilled water for 15 s, gentle air dry for 5 s 2. Apply Single bond universal plus to the dentin surface and rub it for 20 s: gentle air dry for 5 s without light curing 3. Apply cement with an auto-mix tip, light cure for 10 s
PSA	SA	Panavia SA Luting Multi	1. Apply with an auto-mix tip (provided by manufacturer), light cure for 10 s
	SE	Clearfil Tri-s Bond Universal Quick, Panavia SA Luting Multi	1. Apply Clearfil tri-s bond universal quick to the dentin surface and rub it for 5 s: gentle air dry for 5 s without light curing 2. Apply cement with an auto-mix tip, light cure for 10 s
	ER	37% Phosphoric acid etchant gel, Clearfil Tri-s Bond Universal Quick, Panavia SA Luting Multi	1. Apply etchant gel to dentin for 15 s; rinsed with distilled water for 15 s, gentle air dry for 5 s 2. Apply Clearfil tri-s bond universal quick to the dentin surface and rub it for 5 s: gentle air dry for 5 s without light curing 3. Apply cement with an auto-mix tip, light cure for 10 s

Abbreviation: RXU, Rely X Universal; PSA, Panavia SA Luting Multi; SA, Self-adhesive; SE, Self-etch; ER, Etch-and-rinse

The specimens were cemented on the dentin surface under a constant seating force of 500 g for 20 s, and photoactivation was performed for 2 s for tack curing with a light curing unit (Blue phase G2, Ivoclar Vivadent, Schann, Liechtenstein). The excess cement was carefully removed and light-cured following the manufacturer’s instructions. After the cementation procedure, the specimens were kept in a humidified atmosphere at 37 °C for 24 h.

### Shear bond strength test

After the storage period, each specimen was mounted onto a metal holder and placed in the universal testing machine (Instron model 5585 H, Instron Corp, Canton, MA, USA). The shear bond strength (SBS) of the bonded specimens was measured by loading them to failure with a chisel at a crosshead speed of 1.0 mm/min. SBS values were calculated in megapascals (MPa) from the maximum stress (N) at failure divided by the specimen’s surface area. Means and standard deviations were recorded for each group.

### Failure mode analysis

After the SBS test, the failure analysis of all specimens was performed using a microscope at 40x magnification (DP22, Olympus Co., Tokyo, Japan). The mode of failure was classified into three types [24]: (1) cohesive failure in dentin (fractures occurring exclusively within the dentin) (2) mixed failure (partial cement fractures and partial dentin exposure) and (3) adhesive failure (fracture sites entirely located between the resin cement and dentin surface).

### Cement-dentin interface observation

For the cement-dentin interface observation, the dentin surfaces were prepared in the same manner as for the bond strength test. The ceramic discs were bonded to specimens under a constant seating force of 500 g for 20 s, with the specific resin cement for each experimental group. After 24 h of storage in water, an approximately 1-mm thick ceramic-dentin slab was sectioned in the bucco-lingual dimension using a low-speed diamond saw. The slabs were embedded in epoxy resin and polished with a series of #600-, 800-, 1,000-, 1,200-, and 1,500-grit SiC paper under running water, followed by diamond pastes with particle sizes of 6, 3, 1, and 0.25 μm [19]. The specimens were cleaned in an ultrasonic machine for 5 min after polishing with each diamond paste. Subsequently, the specimens were treated with 10% phosphoric acid for 3 s, followed by immersion in 5.25% sodium hypochlorite for 5 min. After drying for 24 h at 37 °C, the embedded specimens were mounted on aluminum stubs. Finally, the specimens were coated with palladium using a K500X Sputter coater (SPI Supplies, West Chester, PA, USA) and observed using an SEM (JSM 6610LV, JEOL Inc., Peabody, MA, USA).

### Dentin surface characteristics before cementation

Four teeth were used to evaluate the etching effects of the adhesive strategies before resin cement application. Dentin discs were prepared using a diamond saw, with each surface divided into two halves prepared by super-fine and coarse diamond burs. The discs were treated according to the adhesive strategies (SA: no treatment, SE: universal adhesive, ER: phosphoric acid etched dentin). In the SE group, adhesive was applied following the manufacturers’ instructions, then removed by immersing the discs in 100% acetone for 60 s. The samples were dehydrated with a graded series of ethanol and dried with hexamethyldisilazane [20]. The specimens were coated as previously described and observe using SEM at 5,000X. (JSM 6610LV, JEOL Inc., Peabody, MA, USA).

### Statistical analysis

The SBS (MPa) was calculated by dividing the applied force (N) at the time of fracture by the bonded area (mm^2^). Data normality and homogeneity of variance was assessed using the Shapiro-wilk test and Levene’s test, respectively. Data analysis was conducted using three-way ANOVA and Game-Howell tests. The statistical analyses were performed using SPSS 29.0 (IBM Corp., Armonk, NY, USA) at a 95% confidence level.

## Results

### Dentin shear bond strength (SBS)

The data for all groups were normally distributed. One specimen from the RXU and PSA groups, cemented in SA mode on coarse diamond bur-prepared dentin, was a pre-test failure. These failures were included in the statistical analysis with a bond strength value of 0 MPa. Three-way ANOVA revealed a significant effect of adhesive strategies (*p* < 0.001) and type of UC (*p* < 0.001) on dentin SBS values, but not for the type of dental bur used for tooth preparation (*p* > 0.05).

The SBS is presented in Table 3. The highest bond strength was observed in the ER group, followed by the SE and SA groups. In the RXU group, the ER mode demonstrated a significantly higher bond strength compared with the SE and SA modes when a coarse diamond bur was used. Whereas, ER and SE demonstrated significantly higher SBS than SA when a super-fine diamond bur was used. In contrast, a significant effect in the PSA group was found only in coarse diamond burs, in which ER demonstrated a higher SBS than SA. Comparing resin cement systems, RXU in SE modes showed a higher SBS than PSA, particularly in dentin prepared with a coarse diamond bur.

**Table 3:** Dentin SBS values (MPa) in different resin luting systems in different etching modes

Resin Cement	burs	Adhesive strategies		
		SA	SE	ER
		Means (±SD)	Means (±SD)	Means (±SD)
RXU	super-fine	8.6642 (±3.364) ^Ba^	15.2602 (±4.316) ^Aa^	23.2205(±9.705) ^Aa^
	coarse	7.5962 (±3.987) ^Ca^	13.1022 (±2.631) ^Ba^	22.1185 (±6.457) ^Aa^
PSA	super-fine	7.0540 (±2.441) ^Aa^	9.4564 (±6.890) ^Aab^	12.4905 (±6.664) ^Aa^
	coarse	4.8686 (±3.107) ^Ba^	7.1771 (±3.709) ^ABb^	13.3179 (±3.649) ^Aa^

Same Upper-case letter in horizontal rows indicates no difference at 5% significance level. Same lower case in vertical columns indicates no difference at 5% significance level., RXU, RelyX Universal; PSA, Panavia SA luting multi; SA, self-adhesive mode; SE, Self-etch mode; ER, Etch-&-rinse mode.

### Failure mode

**Fig. 1:**
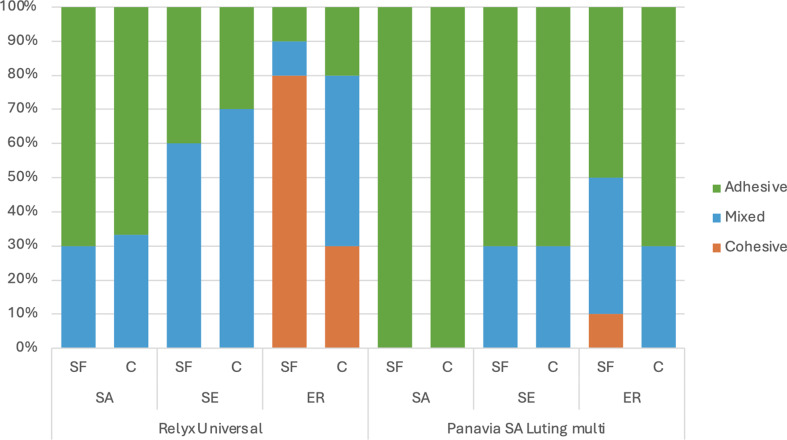
Incidence of failure modes (%) of two universal resin cements bonded with various adhesive strategies and diamond burs, RXU, RelyX Universal; PSA, Panavia SA luting multi; SA, self-adhesive mode; SE, self-etch; ER, Etch-and-rinse mode; SF, super-fine diamond burs; C, coarse diamond burs

The distribution of the failure modes is shown in Fig. 1, with adhesive failure being the predominant mode, followed by mixed failure. The ER mode exhibited a higher incidence of cohesive failure, especially when super-fine diamond burs were used. In contrast, the SA mode had predominantly adhesive failures, particularly with PSA.

### SEM analysis

**Fig. 2:**
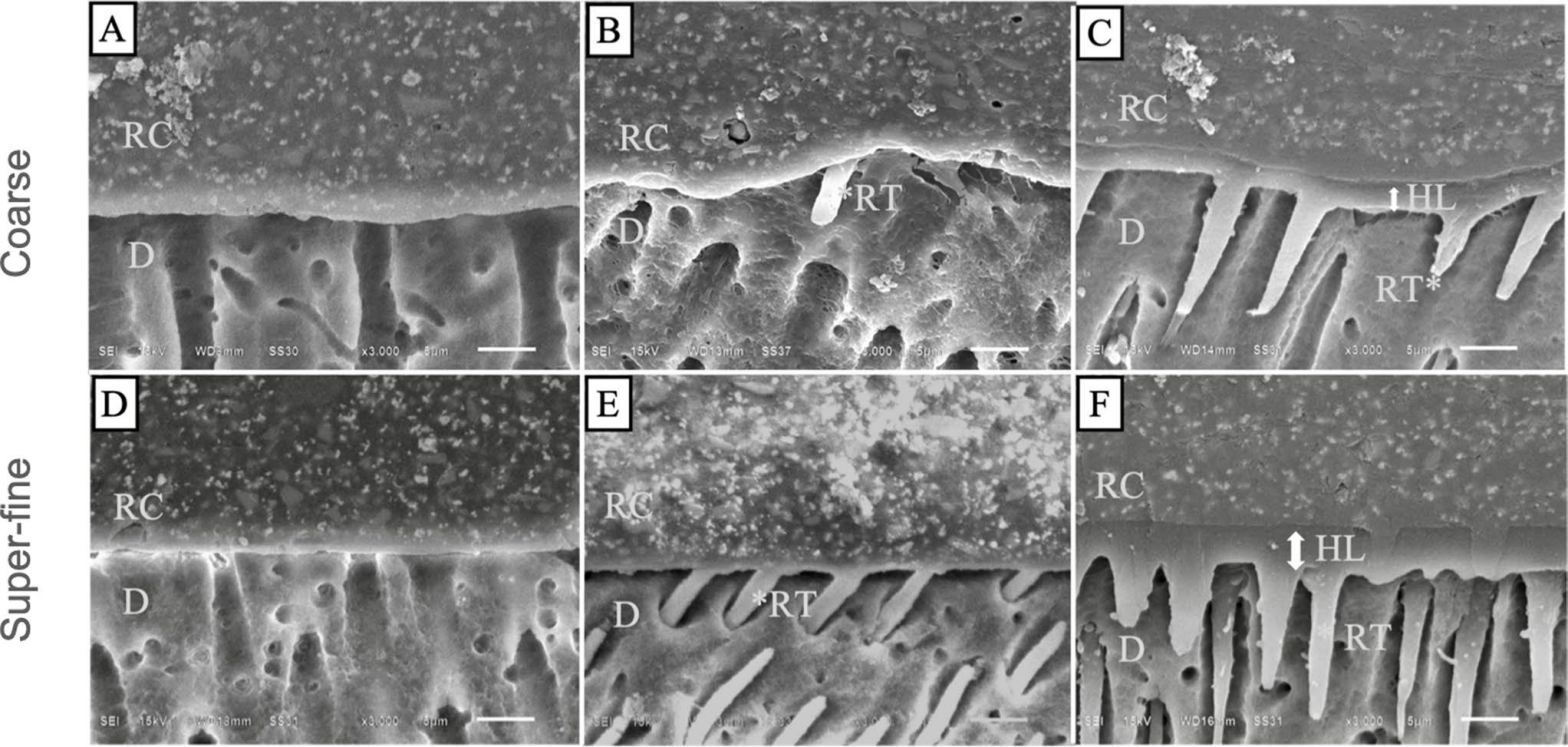
Representative SEM images of the cement-dentin interfaces produced by RXU at 3,000x magnification; (**A**-**C**) dentin prepared with a coarse diamond bur, (**A**) Self-adhesive mode, (**B**) Self-etch mode, (**C**) Etch-and-rinse mode, (**D**-**F**) dentin prepared with a super-fine diamond bur, (**D**) Self-adhesive mode, (**E**) Self-etch mode, (**F**) Etch-and-rinse mode. Abbreviation: D, dentin; RC, resin cement; RT, resin tag; HL, hybrid layer

**Fig. 3:**
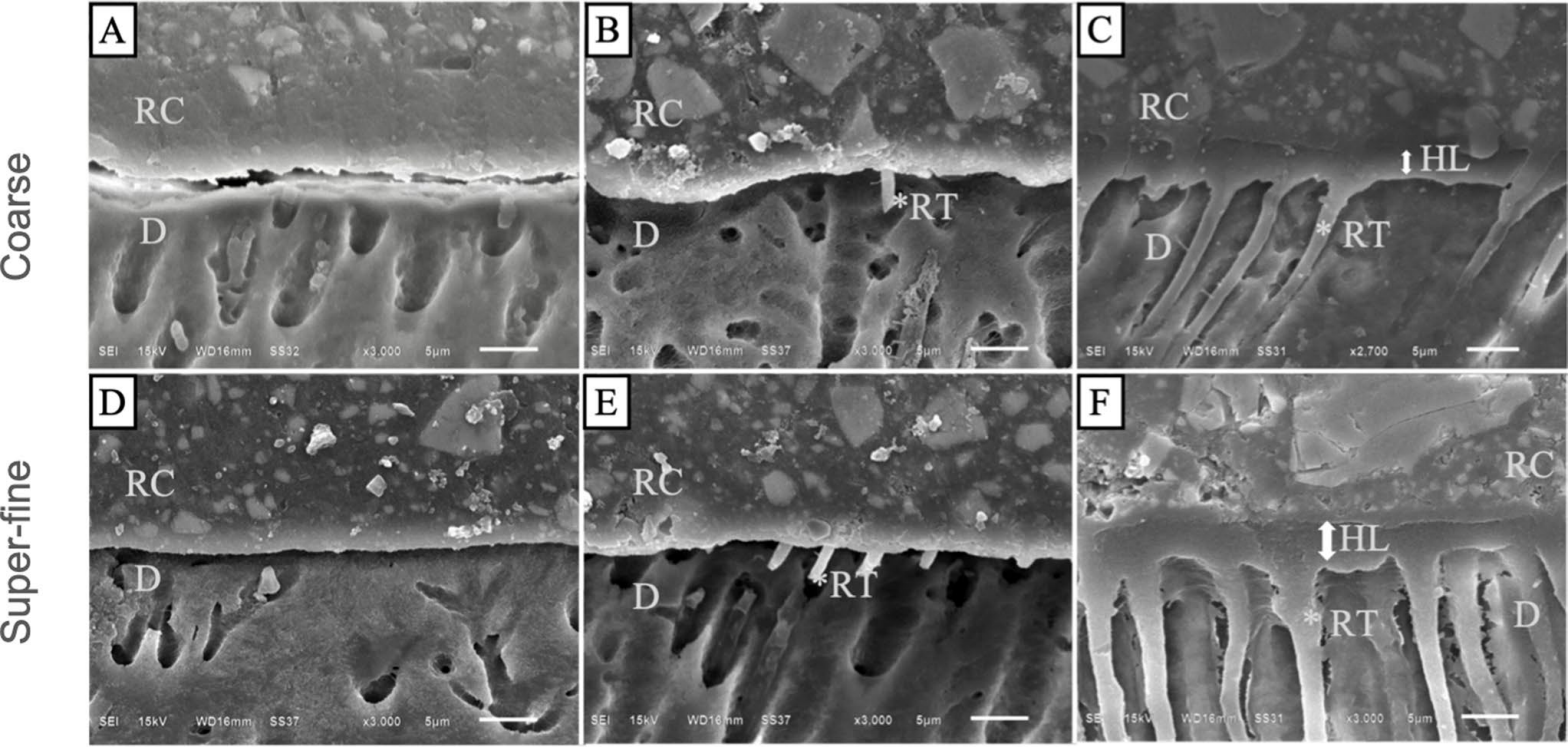
Representative SEM images of the cement-dentin interfaces produced by PSA at 3000x magnification; (**A**-**C**) dentin prepared with a coarse diamond bur, (**A**) Self-adhesive mode, (**B**) Self-etch mode, (**C**) Etch-and-rinse mode, (**D**-**F**) dentin prepared with a super-fine diamond bur, (**D**) Self-adhesive mode, (**E**) Self-etch mode, (**F**) Etch-and-rinse mode. Abbreviation: D, dentin; RC, resin cement; RT, resin tag; HL, hybrid layer

Representative SEM images of the cement-dentin interface of RXU and PSA are demonstrated in Figs. 2 and 3, respectively. In the SA mode, hybrid layers and resin tags were not detected in either cement (Figs. 2A and D and 3A, and 3D). In the SE mode, the resin tags were cylindrical in shape, with RXU showing deeper penetration (Fig. 2B and E). In contrast, resin tags were poorly formed in PSA (Fig. 3B and E). In the ER mode, approximately 2–3 micron-thick hybrid layers were observed (Figs. 2C and F and 3C, and 3F). The resin tags in ER mode were conical in shape, longer, and more abundant compared with the other adhesive strategies.

### Dentin surface characteristic before cementation

**Fig. 4:**
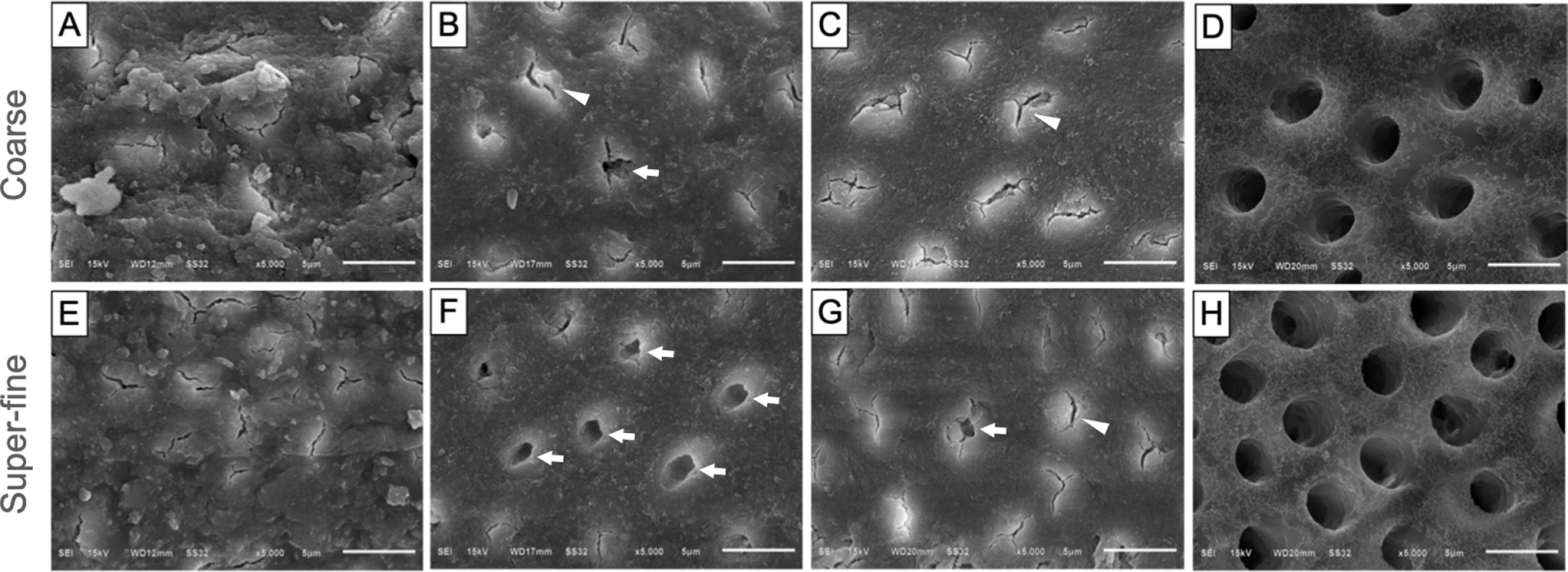
Representative SEM images of the demineralization effect of different adhesive strategies at 5,000x magnification. (**A**-**D**) Dentin prepared with a coarse diamond bur: (**A**) No treatment; (**B**) SBUP applied in self-etch mode; (**C**) CBQ applied in self-etch mode; (**D**) phosphoric acid etched dentin. (**E**-**H**) Dentin prepared with a super-fine diamond bur: (**E**) No treatment; (**F**) SBUP applied in self-etch mode; (**G**) CBQ applied in self-etch mode; (**H**) phosphoric acid etched dentin. Block arrows indicate opened dentinal tubules; Arrowheads indicate dentinal tubules with smear plugs. Abbreviations: SBUP, Scotchbond^™^ Universal Plus; CBQ, Clearfil^™^ Tri-S Universal Bond Quick

Representative SEM images of the dentin surface before cementation, grouped by adhesive strategies, are shown in Fig. 4. In the SA mode (Fig. 4A and D), the entire surface was covered with smear layers for both bur types. In the SE mode, the smear layers were partially dissolved. Most of the smear plugs were occluded in the dentinal tubules (Fig. 4B, C, F and G). More opened dentinal tubules were observed in the Scotchbond^™^ Universal Plus (SBUP) group applied on the super-fine diamond bur prepared dentin (Fig. 4F). The smear layers and plugs as well as peritubular dentin were completely removed in the phosphoric acid etched dentin (Fig. 4D and H), exposing wider dentinal tubules.

## Discussion

The present study evaluated the effect of adhesive strategies, surface preparation with different diamond burs and type of UC on the bond strength of UCs to dentin. The results indicated a significant effect of adhesive strategies for both UCs. Therefore, the first null hypothesis was not accepted. The surface preparation with super-fine diamond burs and coarse diamond burs demonstrated similar bond strength. Thus, the second null hypothesis was accepted. A significantly higher bond strength for RXU was observed when cemented in the ER and SE modes. Therefore, the third null hypothesis was not accepted.

The results of this study revealed that using UCs alone demonstrated the lowest bond strength, whereas SBS significantly increased when combined with a universal adhesive (Table 3). This is in agreement with previous studies that reported that using UCs alone is not effective in completely demineralizing or dissolving the smear layer, resulting in no decalcification or infiltration of resin into the dentin [25–27]. This is also supported by our cement-dentin interface observations (Figs. 2 and 3). Using UC in SA mode demonstrated an intimate adaptation of the cement, but no formation of a hybrid layer or resin tags (Figs. 2A and D and 3A, and 3D). Additionally, the lone pre-test failure occurred when the dentin was prepared with a coarse diamond bur and cemented in SA mode.

A significant increase in SBS was observed for RXU when adhesively cemented (ER and SE). However, for PSA, a significantly higher SBS was observed only in the coarse diamond bur group in ER mode. In ER mode, the application of phosphoric acid assists in completely removing the smear layer, exposing collagen fibers, and creating micro-mechanical interlocking by demineralizing the underlying dentin [5]. This allows the adhesive to infiltrate the dentin, forming resin tags and a hybrid layer. This is supported by the failure mode analysis and cement-dentin interface observations. The higher percentage of cohesive and mixed failures were found in ER mode (Fig. 2), especially with RXU, indicating a strong bond. Furthermore, resin tags and a hybrid layer were detected in ER mode (Fig. 3). In contrast, using UC in SE mode had a significantly higher bond strength than SA mode, however a significant effect was observed only in RXU and PSA with coarse diamond burs. The higher bond strength of SE mode is consistent with previous studies [11–12, 28]. This could be due to the application of universal adhesive in SE mode that enhances smear layer removal, increases surface wetting and promotes resin penetration (compare Fig. 2A, B, D and E).

The coarse and super-fine diamond bur preparations did not differentially affect the results in this study. This can be separately explained according to the adhesive strategies. For ER mode, phosphoric acid completely removes the smear layer and demineralizes the underlying dentin, increasing surface roughness. These results are supported by the SEM observations (Fig. 4), which demonstrated the etching efficacy of the ER mode (Fig. 4D and H), revealing visible dentinal tubules. Therefore, the effect of bur preparation was not significant in this case, as supported by previous studies. [29–30]

It was reported that the bond strength of self-etch adhesives was affected by different diamond bur preparations [18–20]. Therefore, the thicker smear layer produced by coarse burs was more difficult to dissolve [14, 16], negatively impacting the bond strength [16, 18–20, 29]. However, the SBS of UC cemented in SE mode with super-fine and coarse diamond burs were similar (Table 3). The effect of surface roughness and the smear layer can paradoxically influence the bond strength. The increased roughness may enhance the surface area for bonding, whereas the smear layer may interfere with the bonding process. It was reported the surface area increased from 0.19 to 2.95% whereas, the thicknesses of the smear layer were 0.95 and 2.24 micron with an extra fine and coarse diamond bur, respectively [19]. This reasoning may explain why the SBS of UCs in SE mode was not affected by diamond burs.

Similarly, the effect of diamond bur preparation was not observed in SA mode for both cements. This could be attributed to material composition combined with seating pressure. The UCs used in this study are newly developed. RXU incorporates an amphiphilic redox initiator system, which forms a firm cross-linking polymer network to increase the conversion rate within the hydrophilic cement, and a new filler architecture designed to optimize the material’s rheological characteristics. The addition of HEMA in the resin cement might facilitate the wettability of the dentin surface. Additionally, the pH of the cements was reduced from 2.125 to 1.7 for RXU [31] and from 3.025 to 2.8 for PSA [31], which could enhance their ability to dissolve the smear layer. Furthermore, the seating pressure during ceramic cementation promotes good adaptation of the cement to dentin. This could facilitate the interaction between the functional monomer in the cements with the smear layer-covered dentin surface. [32–33] These could be the reasons why the bond strengths of both UCs were unaffected by the smear layer created by the different diamond burs.

The overall bond strength of RXU was higher than that of PSA. However, a significant effect was observed when the cements were bonded in SE modes with coarse diamond bur prepared dentin, which can be explained by several factors. The difference in adhesive application times was due to the different manufacturers’ instructions. PSA, which uses Clearfil™ Tri-S Bond Universal Quick (CBQ) and requires “no waiting time” according to the manufacturer’s instructions [34]. In this study, a 5-sec application was used [35], which is shorter than the 20-sec application required for SBUP in the RXU group [36]. The shorter adhesive application time might lead to a lower bond strength due to insufficient solvent evaporation and less effective smear layer demineralization [37–38]. This is supported by the different demineralization effect between SBUP and CBQ (Fig. 4). SBUP applied in SE mode partially removed the smear layer, revealing dentinal tubules, especially in superfine-prepared dentin (Fig. 4F). In contrast, other SE groups showed limited visibility of the tubules (Fig. 4B, C and G), particularly in coarse prepared dentin (Fig. 4C). This suggests that SBUP may have a greater potential to remove the smear layers than CBQ. This is in line with a previous study showing inferior bond strength due to shorter application time, especially for CBQ [39].

The functional monomer contained in the tested adhesives should also be discussed. In this study, the adhesive tested was SBUP, containing multiple functional monomers (10-MDP and Vitrebond co-polymer), while CBQ contains only 10-MDP. The presence of multiple functional monomers likely enhances bonding by interacting more effectively with the compact bur-cut smear layer [37–38], potentially explaining why RXU demonstrated a higher bond strength than PSA. The more aggressive etching pattern and deeper resin penetration was observed with SBUP compared with those of CBQ, despite the higher pH of SBUP (SBUP: pH = 2.7, CBQ: pH = 2.3 [31]). Moreover, the differences in the composition of the resin cement and adhesive systems could play a role in the bonding performance. PSA cement is mainly composed of the Bis-GMA resin monomer, while RXU is Bis-GMA free and uses diurethane dimethacrylate (DUDMA) as a co-monomer [36]. DUDMA reduces polymerization stress and increases hydrophobicity without compromising the material’s mechanical properties [40].

There are some limitations in this study. The teeth were sectioned perpendicular to their long axis, resulting in a bonding surface mostly perpendicular to the dentinal tubules. However, in crown preparation, which exposes the dentinal tubules in wider areas and different directions, the effect of dentinal tubule orientation should be considered [41]. Moreover, the effects of simulated aging were not assessed. Cyclic loading and thermocycling are commonly used and have been reported to significantly reduce bond strength by 31.71% and 30.57%, respectively, in the older resin cement generation [42]. In contrast, the results for UCs after thermocycling are less consistent. No influence of 10,000 cycles of thermocycling was observed [43], whereas a negative impact of thermocycling was observed in another study [31].

Overall, our findings highlight the intricate relationship between resin luting systems, adhesive strategies, and dental bur preparations in determining bond strength. Further investigation is warranted to explore the influence of different dental bur preparations and the use of adhesive bonding agents with various curing modes and long-term bonding performance. This is essential for fully understanding the working mechanisms and optimizing the efficiency of universal resin cements to achieve optimal performance.

## Conclusion

Within the limitations of this study, it was concluded that:

1. The bonding performance of UCs is influenced by the adhesive strategies used and the resin cement system. The etch-and-rinse mode is recommended, as it provides the highest dentin bond strength and the most favorable fracture modes.
2. Different particle sizes of diamond bur preparations did not significantly affect the overall bonding performance of UCs.
3. The bonding performance of UCs is material-dependent, with RelyX Universal demonstrating higher bond strength than Panavia SA Luting Multi.

## Data Availability

No datasets were generated or analysed during the current study.

## References

[1] Manso AP, Carvalho RM (2017) Dental Cements for Luting and Bonding Restorations: Self-Adhesive Resin Cements. Dent Clin North Am 61(4):821–834. 10.1016/j.cden.2017.06.006 28886770 10.1016/j.cden.2017.06.006

[2] Nakamura T, Wakabayashi K, Kinuta S, Nishida H, Miyamae M, Yatani H (2010) Mechanical properties of new self-adhesive resin-based cement. J Prosthodont Res 54(2):59–64. 10.1016/j.jpor.2009.09.004 19879828 10.1016/j.jpor.2009.09.004

[3] Hikita K, Van Meerbeek B, De Munck J, Ikeda T, Van Landuyt K, Maida T, Lambrechts P, Peumans M (2007) Bonding effectiveness of adhesive luting agents to enamel and dentin. Dent Mater 23(1):71–80. 10.1016/j.dental.2005.12.002 16426673 10.1016/j.dental.2005.12.002

[4] Radovic I, Monticelli F, Goracci C, Vulicevic ZR, Ferrari M (2008) Self-adhesive resin cements: a literature review. J Adhes Dent 10(4):251–258 18792695

[5] Duarte S, Sartori N, Sadan A, Phark J-H (2011) Adhesive resin cements for bonding esthetic restorations: a review. Quintessence Dent Technol 34:40–66

[6] Sousa SJL, Poubel D, Rezende L, Almeida FT, de Toledo IP, Garcia FCP (2020) Early clinical performance of resin cements in glass-ceramic posterior restorations in adult vital teeth: A systematic review and meta-analysis. J Prosthet Dent 123(1):61–70. 10.1016/j.prosdent.2018.12.006 30982625 10.1016/j.prosdent.2018.12.006

[7] Pfister JL, Federlin M, Hiller KA, Schmalz G, Buchalla W, Cieplik F, Scholz KJ (2023) Randomized Clinical Split-Mouth Study on Partial Ceramic Crowns Luted with a Self-adhesive Resin Cement with or without Selective Enamel Etching: Long-Term Results after 15 Years. J Adhes Dent 25(1):177–186. 10.3290/j.jad.b4478817 37800873 10.3290/j.jad.b4478817 PMC11734261

[8] Miotti LL, Follak AC, Montagner AF, Pozzobon RT, da Silveira BL, Susin AH (2020) Is Conventional Resin Cement Adhesive Performance to Dentin Better Than Self-adhesive? A Systematic Review and Meta-Analysis of Laboratory Studies. Oper Dent 45(5):484–495. 10.2341/19-153-L 32101496 10.2341/19-153-L

[9] Alghauli MA, Alqutaibi AY, Wille S, Kern M (2023) Clinical reliability of self-adhesive luting resins compared to other adhesive procedures: A systematic review and meta-analysis. J Dent 129:104394. 10.1016/j.jdent.2022.104394 36566829 10.1016/j.jdent.2022.104394

[10] Atalay C, Koc Vural U, Miletic I, Gurgan S (2022) Shear bond strengths of two newly marketed self-adhesive resin cements to different substrates: A light and scanning electron microscopy evaluation. Microsc Res Tech 85(5):1694–1702. 10.1002/jemt.24031 34921572 10.1002/jemt.24031

[11] Rohr N, Martin S, Zitzmann NU, Fischer J (2022) A comprehensive in vitro study on the performance of two different strategies to simplify adhesive bonding. J Esthet Restor Dent 34(5):833–842. 10.1111/jerd.12903 35305288 10.1111/jerd.12903 PMC9543337

[12] Breschi L, Josic U, Maravic T, Mancuso E, Del Bianco F, Baldissara P, Mazzoni A, Mazzitelli C (2023) Selective adhesive luting: A novel technique for improving adhesion achieved by universal resin cements. J Esthet Restor Dent. 10.1111/jerd.13037 36971211 10.1111/jerd.13037

[13] Maravic T, Mazzitelli C, Mancuso E, Del Bianco F, Josic U, Cadenaro M, Breschi L, Mazzoni A (2023) Resin composite cements: Current status and a novel classification proposal. J Esthet Restor Dent. 10.1111/jerd.13036 36924395 10.1111/jerd.13036

[14] Saikaew P, Matsumoto M, Sattabanasuk V, Harnirattisai C, Carvalho RM, Sano H (2020) Ultra-morphological characteristics of dentin surfaces after different preparations and treatments. Eur J Oral Sci 128(3):246–254. 10.1111/eos.12698 32396258 10.1111/eos.12698

[15] Oliveira AC, Lima LM, Pizzolitto AC, Santos-Pinto L (2010) Evaluation of the smear layer and hybrid layer in noncarious and carious dentin prepared by air abrasion system and diamond tips. Microsc Res Tech 73(6):597–605. 10.1002/jemt.20798 19941294 10.1002/jemt.20798

[16] Trivedi P, Dube M, Pandya M, Sonigra H, Vachhani K, Attur K (2014) Effect of different burs on the topography of smear layer formation on the dentinal surface: a scanning electron microscope study. J Contemp Dent Pract 15(2):161–164. 10.5005/jp-journals-10024-1507 25095836 10.5005/jp-journals-10024-1507

[17] Cerqueira L, Costa A, Spohr A, Miyashita E, Miranzi B, Calabrez-Filho S, Sobrinho L, Borges G (2018) Effect of Dentin Preparation Mode on the Bond Strength Between Human Dentin and Different Resin Cements. Braz Dent J 29:268–274. 10.1590/0103-6440201801809 29972453 10.1590/0103-6440201801809

[18] Ren L, Li M, Pan Y, Meng X (2018) Influence of Polishing Methods on the Bonding Effectiveness and Durability of Different Resin Cements to Dentin. Biomed Res Int. 10.1155/2018/9189354 29682570 10.1155/2018/9189354 PMC5851321

[19] Saikaew P, Senawongse P, Chowdhury AFMA, Sano H, Harnirattisai C (2018) Effect of smear layer and surface roughness on resin-dentin bond strength of self-etching adhesives. Dent Mater J 37(6):973–980. 10.4012/dmj.2017-349 30135339 10.4012/dmj.2017-349

[20] Saikaew P, Chowdhury A, Matsumoto M, Carvalho R, Sano H (2020) Effects of Double Application of Resin Cement Primers and Different Diamond Burs on Cement-Dentin Bond Strength. J Adhes Dent 22(3):311–320. 10.3290/j.jad.a44554 32435771 10.3290/j.jad.a44554

[21] Faul F, Erdfelder E, Lang AG, Buchner A (2007) G*Power 3: a flexible statistical power analysis program for the social, behavioral, and biomedical sciences. Behav Res Methods 39(2):175–191. 10.3758/bf03193146 17695343 10.3758/bf03193146

[22] Siriporananon C, Senawongse P, Sattabanasuk V, Srimaneekarn N, Sano H, Saikaew P (2021) Effects of dentin surface preparations on bonding of self-etching adhesives under simulated pulpal pressure. Restor Dent Endod 47(1):e4–e. 10.5395/rde.2022.47.e4 35284320 10.5395/rde.2022.47.e4 PMC8891469

[23] Yoshihara K, Nagaoka N, Maruo Y, Nishigawa G, Yoshida Y, Van Meerbeek B (2020) Silane-coupling effect of a silane-containing self-adhesive composite cement. Dent Mater 36(7):914–926. https://doi.org/https://doi.org/10.1016/j.dental.2020.04.014 32473833 10.1016/j.dental.2020.04.014

[24] Scherrer SS, Cesar PF, Swain MV (2010) Direct comparison of the bond strength results of the different test methods: a critical literature review. Dent Mater 26(2):e78–93. 10.1016/j.dental.2009.12.002 20060160 10.1016/j.dental.2009.12.002

[25] De Munck J, Vargas M, Van Landuyt K, Hikita K, Lambrechts P, Van Meerbeek B (2004) Bonding of an auto-adhesive luting material to enamel and dentin. Dent Mater 20(10):963–971. 10.1016/j.dental.2004.03.002 15501325 10.1016/j.dental.2004.03.002

[26] Monticelli F, Osorio R, Mazzitelli C, Ferrari M, Toledano M (2008) Limited decalcification/diffusion of self-adhesive cements into dentin. J Dent Res 87(10):974–979. 10.1177/154405910808701012 18809754 10.1177/154405910808701012

[27] Mazzitelli C, Monticelli F, Osorio R, Casucci A, Toledano M, Ferrari M (2008) Effect of simulated pulpal pressure on self-adhesive cements bonding to dentin. Dent Mater 24(9):1156–1163. 10.1016/j.dental.2008.01.005 18295325 10.1016/j.dental.2008.01.005

[28] Andrews EK, Gedge JL, Vandewalle KS (2023) Bond Strength of a Novel Universal Resin Cement to Dentin with or without an Adhesive Bonding Agent: An In Vitro Study. J Contemp Dent Pract 24(10):725–728. 10.5005/jp-journals-10024-3568 38152902 10.5005/jp-journals-10024-3568

[29] Oliveira SS, Pugach MK, Hilton JF, Watanabe LG, Marshall SJ, Marshall GW (2003) Jr. The influence of the dentin smear layer on adhesion: a self-etching primer vs. a total-etch system. Dent Mater 19(8):758–767. 10.1016/s0109-5641(03)00023-x 14511734 10.1016/s0109-5641(03)00023-x

[30] Hosoya Y, Shinkawa H, Suefiji C, Nozaka K, Garcia-Godoy F (2004) Effects of diamond bur particle size on dentin bond strength. Am J Dent 17(5):359–364 15580682

[31] Watanabe S, Takamizawa T, Hayashi K, Aoki R, Barkmeier WW, Latta MA, Watanabe H, Miyazaki M (2024) Comparing Various Resin Luting Cement Systems in Different Etching Modes Through Bond Durability and Morphological Features. Oper Dent 49(2):231–244. 10.2341/23-096-L 38349845 10.2341/23-096-L

[32] Chieffi N, Chersoni S, Papacchini F, Vano M, Goracci C, Davidson CL, Tay FR, Ferrari M (2007) The effect of application sustained seating pressure on adhesive luting procedure. Dent Mater 23(2):159–164. 10.1016/j.dental.2006.01.006 16494935 10.1016/j.dental.2006.01.006

[33] Goracci C, Cury AH, Cantoro A, Papacchini F, Tay FR, Ferrari M (2006) Microtensile bond strength and interfacial properties of self-etching and self-adhesive resin cements used to lute composite onlays under different seating forces. J Adhes Dent 8(5):327–335 17080881

[34] Kuraray Noritake Dental Inc Technical Information: CLEARFIL™ TRI-S BOND Universal Quick, [ https://www.kuraraynoritake.eu/media/pdfs/clearfil-universal-bond-quick-scientific-product-information-en.pdf

[35] Sukprasert N, Harnirattisai C, Senawongse P, Sano H, Saikaew P (2022) Delayed light activation of resin composite affects the bond strength of adhesives under dynamic simulated pulpal pressure. Clin Oral Investig 26(11):6743–6752. 10.1007/s00784-022-04634-3 35876892 10.1007/s00784-022-04634-3

[36] Care 3MO Technical Product Profile: 3M^™^ Scotchbond^™^ Universal Plus Adhesive [ https://multimedia.3m.com/mws/media/1910608O/3m-scotchbond-universal-plus-adhesive-technical-product-profile-us.pdf

[37] Saikaew P, Matsumoto M, Chowdhury A, Carvalho RM, Sano H (2018) Does Shortened Application Time Affect Long-Term Bond Strength of Universal Adhesives to Dentin? Oper Dent 43(5):549–558. 10.2341/17-205-L 29630488 10.2341/17-205-L

[38] Saikaew P, Chowdhury AFMA, Fukuyama M, Kakuda S, Carvalho RM, Sano H (2016) The effect of dentine surface preparation and reduced application time of adhesive on bonding strength. J Dent 47:63–70. 10.1016/j.jdent.2016.02.001 26855030 10.1016/j.jdent.2016.02.001

[39] Ahmed MH, Yoshihara K, Mercelis B, Van Landuyt K, Peumans M, Van Meerbeek B (2020) Quick bonding using a universal adhesive. Clin Oral Investig 24(8):2837–2851. 10.1007/s00784-019-03149-8 31813057 10.1007/s00784-019-03149-8

[40] Fugolin AP, de Paula AB, Dobson A, Huynh V, Consani R, Ferracane JL, Pfeifer CS (2020) Alternative monomer for BisGMA-free resin composites formulations. Dent Mater 36(7):884–892. 10.1016/j.dental.2020.04.009 32402514 10.1016/j.dental.2020.04.009 PMC7305961

[41] Guo J, Wang LP, Zhu J, Yang J, Zhu HS (2018) Impact of Dentinal Tubule Orientation on Dentin Bond Strength. Curr Med Sci 38(4):721–726. 10.1007/s11596-018-1936-8 30128884 10.1007/s11596-018-1936-8

[42] Guarda GB, Correr AB, Goncalves LS, Costa AR, Borges GA, Sinhoreti MA, Correr-Sobrinho L (2013) Effects of surface treatments, thermocycling, and cyclic loading on the bond strength of a resin cement bonded to a lithium disilicate glass ceramic. Oper Dent 38(2):208–217. 10.2341/11-076-L 22856682 10.2341/11-076-L

[43] Muto R, Takamizawa T, Shiratsuchi K, Kasahara Y, Suda S, Watanabe H, Latta MA, Miyazaki M (2024) Influence of luting strategies on dentin bond performance of self-adhesive resin luting cement in combination with a universal adhesive. Clin Oral Investig 28(9):478. 10.1007/s00784-024-05850-9 39122868 10.1007/s00784-024-05850-9

